# A Fire Incident Case at a Radiodiagnostic Center of a Tertiary Care Hospital: Methods for Reduction in Fatality by Smoke Evacuation

**DOI:** 10.7759/cureus.31873

**Published:** 2022-11-24

**Authors:** Mandeep Sachdeva, Raman Sharma, Yadvinder Singh, Deepali Thakur, Vipin Koushal, Ashok Kumar

**Affiliations:** 1 Hospital Administration, Postgraduate Institute of Medical Education and Research, Chandigarh, IND

**Keywords:** compartmentalization, smoke injury, fire safety, pressurization, dilution ventilation, future disaster response, disaster response and preparedness

## Abstract

For the general public, healthcare facilities are always a safe and secure place for treatment. Generally, healthcare institutions are equipped to deal with exterior interruptions, but circumstances brought on by internal risks are more serious and frequently require an emergency evacuation of the facility. An incident happened at the radiodiagnostic setup of a tertiary care institute in North India. This fire incident created panic among staff and patients. At the place of casualty, there were around 150 persons, including staff, patients, and their attendants. Immediately after the confirmation of the fire incident, the fire department and security department took action in the form of fire control and smoke evacuation. Though six fire handling staff required minor emergency services for asphyxia due to smoke inhalation and were cured by oxygen support only, none of the patients was affected due to timely smoke evacuation. Most often, smoke management techniques implemented are compartmentation, pressurization, dilution, ventilation, buoyancy, and airflow. So, we concluded that the step of timely smoke evacuation and preventing the spread of smoke by various methods help to reduce fatality due to smoke. The training programs and mock drills give stakeholders the needed knowledge, skills, and practice they need to safeguard patients and employees.

## Introduction

For the general public, healthcare facilities are always a safe and secure place for treatment. Generally, healthcare institutions are equipped to deal with exterior interruptions, but circumstances brought on by internal risks are more serious and frequently require an emergency evacuation of the facility [1]. Fire incidents are one of the most significant potential risks in healthcare settings out of all hazards. In hospitals, several electrical devices, medical gases, and flammable liquids are frequently used together at one location, and any mistake anywhere has the potential to start a disaster. Since patients are not prepared for such a disaster and do not know how to react, henceforth, in such a crisis, hospital staff has to be well prepared for the safety of the hospitalized patients [2]. Generally, two approaches are used to protect healthcare buildings from fire incidents: the building's design and its usage [3]. Fire safety rules are usually given less attention, primarily during the building design process, with the goal being to meet the bare minimum criteria as per the established building regulations. Moreover, even if such a property is poorly planned, it would be safe from such incidents if a proactive and effective team is in place [4]. On the other hand, a well-designed building is more likely to experience fewer fire safety difficulties throughout its life.

Even though healthcare facilities face many fire hazards, damage caused by smoke rather than flames is often more severe. Instead of burns, smoke inhalation is the primary cause of fatality in fire-related incidents. The greatest cause of illness and mortality in fire victims, particularly in enclosed places, appears to be the inhalation of fire smoke, which contains a complex mixture of gases in addition to carbon monoxide. Smoke inhalation frequently exposes victims to cyanide gas, and in most cases, cyanide is found in the blood of fire victims. This suggests that cyanide may significantly affect smoke inhalation fatalities [5]. Any material subjected to combustion produces particles (solid and liquid) and gases. As per the study by Ebenehi, smoke inhalation is always the main cause of fire-related mortality, accounting for about 75% of all fire-related fatalities. Typically, 57% of fire deaths take place away from the fire's point of origin. The immediate dangers of inhaling smoke are headaches, coughing, shortness of breath, and stinging eyes [6]. Patients with pulmonary signs may experience dyspnea and coughing, while those with cardiac manifestations may have palpitations or chest pain. Generally, after inhaling smoke, a severe inflammatory reaction typically lasts for at least six months. Furthermore, it is well-known that smoke travels via heating, ventilation, and air conditioning (HVAC) systems and to adjoining areas, endangering the patients and the medical personnel stationed there.

Henceforth, this present case scenario has been conceived to determine the smoke-generated impact during fire incidents. These can be used as a guide for future research and to develop policy guidelines relevant to prevention activities.

## Technical report

Our institute is 2250 bedded super-specialty tertiary care teaching hospital in Northern India, with an annual outdoor patient department (OPD) census of approximately twenty-eight lakh and an indoor patient department (IPD) census of around one lakh. Due to this heavy patient load, there is a huge burden on radiological machines, which are working 24 × 7 throughout the year.

Our institute has a very robust firefighting and fire prevention system, a fire control room, advanced firefighting equipment, and trained firefighting officers who are always alert to meet any exigency and ensure strict compliance with statutory and regulatory guidelines of fire safety around the clock. Instruction or guideline boards (in the form of do's and don’ts), emergency fire control numbers and signage have been displayed at different places for the staff and public's awareness.

Fire hydrants and water sprinklers are installed strategically with constant water flow to mitigate any exigency. Regular drills are done to ensure that staff is fully aware of what actions to take in the event of a fire or fire alarm actuating. To mitigate such incidents, the engineering department has been made accountable for scheduled maintenance and a timely audit of all the electric cables and gadgets. All hot jobs are carried out in the institute's working buildings by ensuring adequate fire safety measures.

On Monday, September 20, 2021, at 15:15 hours, there was, as usual, a huge rush in the facility of computed tomography (CT) scan, magnetic resonance imaging (MRI), and ultrasound (USG) in the Department of Radiodiagnosis and Imaging, and at any moment, there are around 40-50 patients or patient attendants in the waiting area, and most of the patients are on wheelchairs or trolleys waiting for the investigations. Suddenly, there was a sparking sound and fire erupted from the MRI unit. And within no time, thick black smoke and the smell of burning material engulfed the whole area, reducing visibility to almost zero. As expected, there was panic and fear among patients and their attendants, and this sudden spread of smoke required an immediate response.

The fire control room was informed, and an immediate evacuation of patients and their attendants to a safer place was started. Within no time, fire personnel and security guards rushed to the site with all necessary firefighting equipment to contain the fire and smoke.

**Figure 1:**
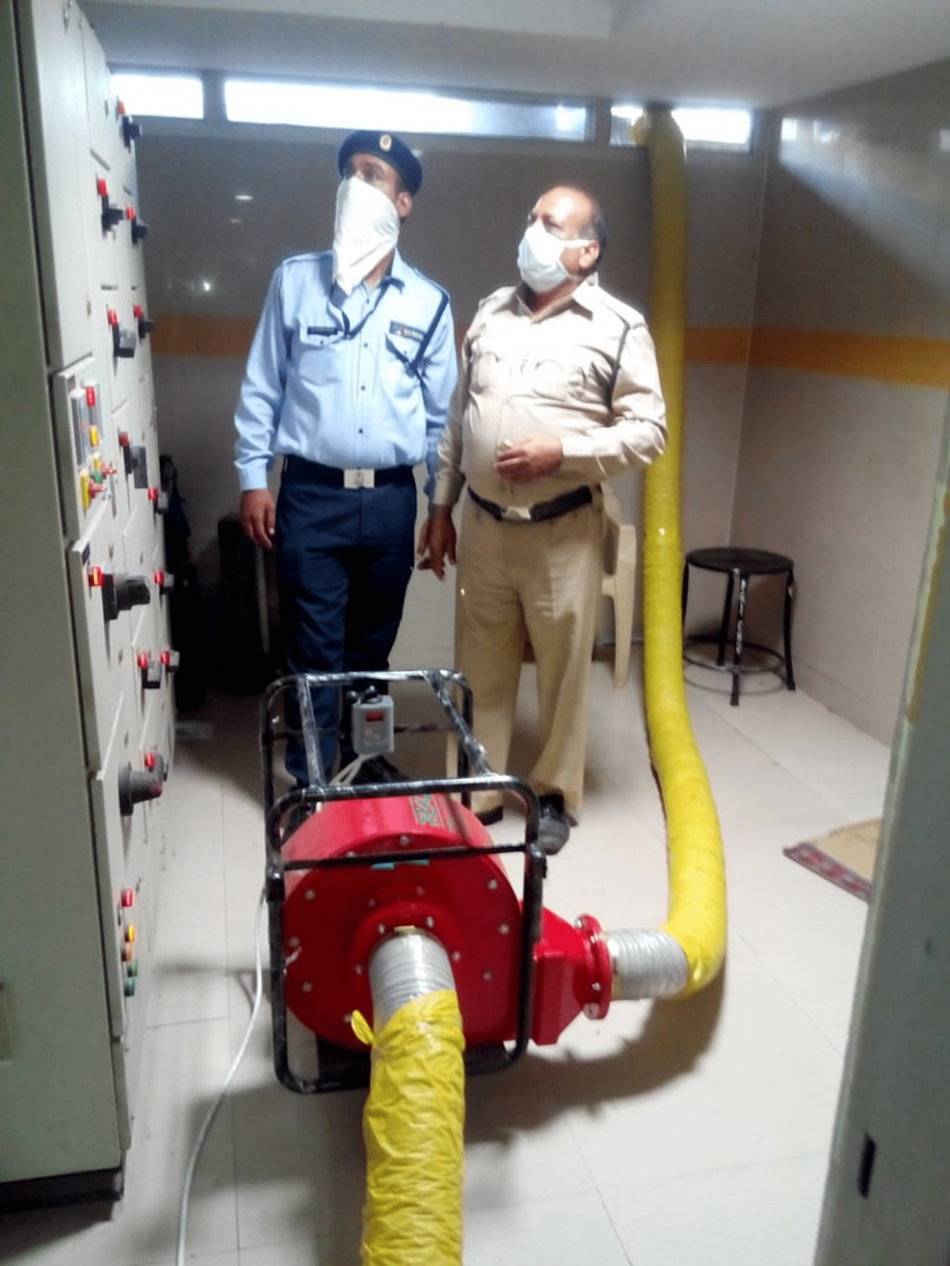
Smoke pump. Fire and security officers are removing smoke from the affected area. Smoke pumps have an inlet for sucking smoke and an outlet for throwing smoke into the open air.

Two smoke exhaust blowers were used to blow out smoke (Figure 1). Glass window panes were opened or broken to ventilate and clear the dense and poisonous smoke from the area. Tables and chairs blocking or obstructing the fire escape route area were also removed. Since ventilation was poor in the area, it took about 50 minutes to clear the smoke from the area.

**Figure 2:**
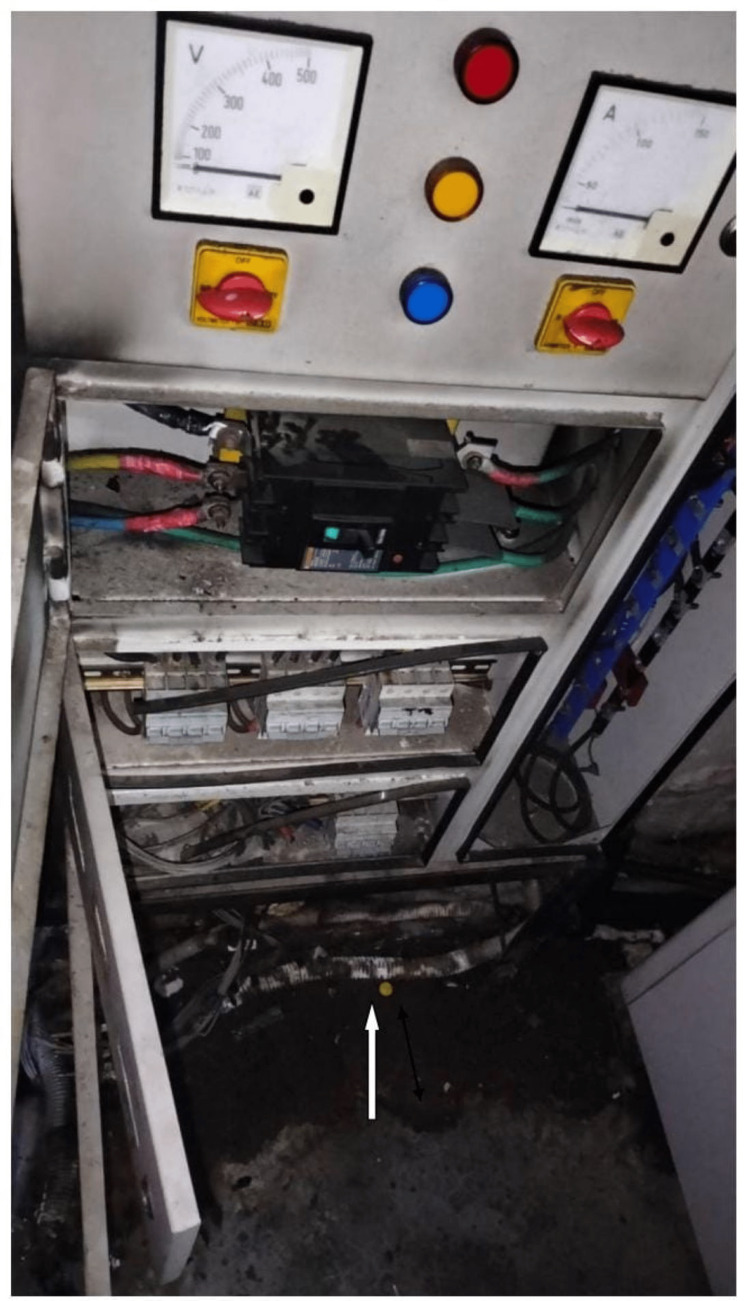
Short circuit of the panel. Short-circuiting of AHU panel wires lead to a fire. AHU: air handling unit.

After doing a root cause analysis, it was found that this fire incident erupted due to a short circuit in the electrical panel of the air handling unit (AHU) supplying the 3T MRI (Figure 2).

The alarm system didn’t get activated, so the AHU didn’t trip down, and smoke spread to the whole area quickly. There was also just one entry and exit gate.

Six firefighting persons got asphyxiated by this poisonous smoke during this fire incident. They were immediately shifted to the emergency room for first aid and required treatment. They were kept under observation overnight and were discharged after recovery from asphyxia. Due to a prompt and proactive response by healthcare workers and timely action by the fire personnel and security officers, the fire was extinguished well in time and no human casualties were reported. It is also pertinent to mention that had the fire not been controlled timely and with prompt action by fire officers and security officers, the impact of this fire incident may have led to an extent as the adjoining room was filled with flammable materials.

## Discussion

Fire occurrences have been identified as one of the most likely internal risks in healthcare settings that call for prompt awareness and remediation. It has been stated that more than three-quarters of all fire deaths are caused by smoke inhalation and that 60% of fire deaths occur beyond the area of the fire's origin. To address the smoke crisis and raise hospital patient safety standards, smoke management, evacuation routes, and relocation areas must be incorporated into the emergency plan requirements.

With the primary goal of ensuring the safety of occupants and property, smoke management is the term used to describe the methods used to passively or actively control the transport of smoke inside the built environment. Most often, the smoke management techniques implemented are compartmentation, pressurization, dilution, ventilation, and buoyancy [7].

Compartmentalization prevents smoke from moving from a fire space to other areas by erecting physical barriers. These barriers include doors, floors, walls, partitions, and smoke dampers. Fire compartmentation is essential for fire containment when a building is divided into several distinct sections or cells. The dividing walls of each cell are packed with specialized materials to stop the fire from spreading from one cell to another.

Protecting "ways of escape" from a building is one of the key advantages of compartmentation. Most large structures are divided into "compartments" that can withstand fire for a set period of time, either internally or externally. This safety barrier provides a window of opportunity for residents to be evacuated, for emergency crews to show up and put out the fire, or for the fire to go out on its own.

Due to compartmentation, hospitals will use a "horizontal phased evacuation" plan in which patients who can leave the building unaided will do so right away, and patients who can't be moved very far will be moved to an adjacent compartment. One of the most important components of fire compartmentation is fire doors. Regular inspections of fire doors are necessary, and any damage or maintenance problems should be resolved immediately. 

High-rise buildings have highly specific needs regarding smoke management because smoke spreads swiftly through them, trapping residents on upper floors and making it harder for them to escape to places with clean air. To stop smoke from spreading quickly throughout the structure through frequent escape routes, including stairways, vents, and elevator shafts, smoke control systems must be installed in addition to firefighting gear.

**Figure 3:**
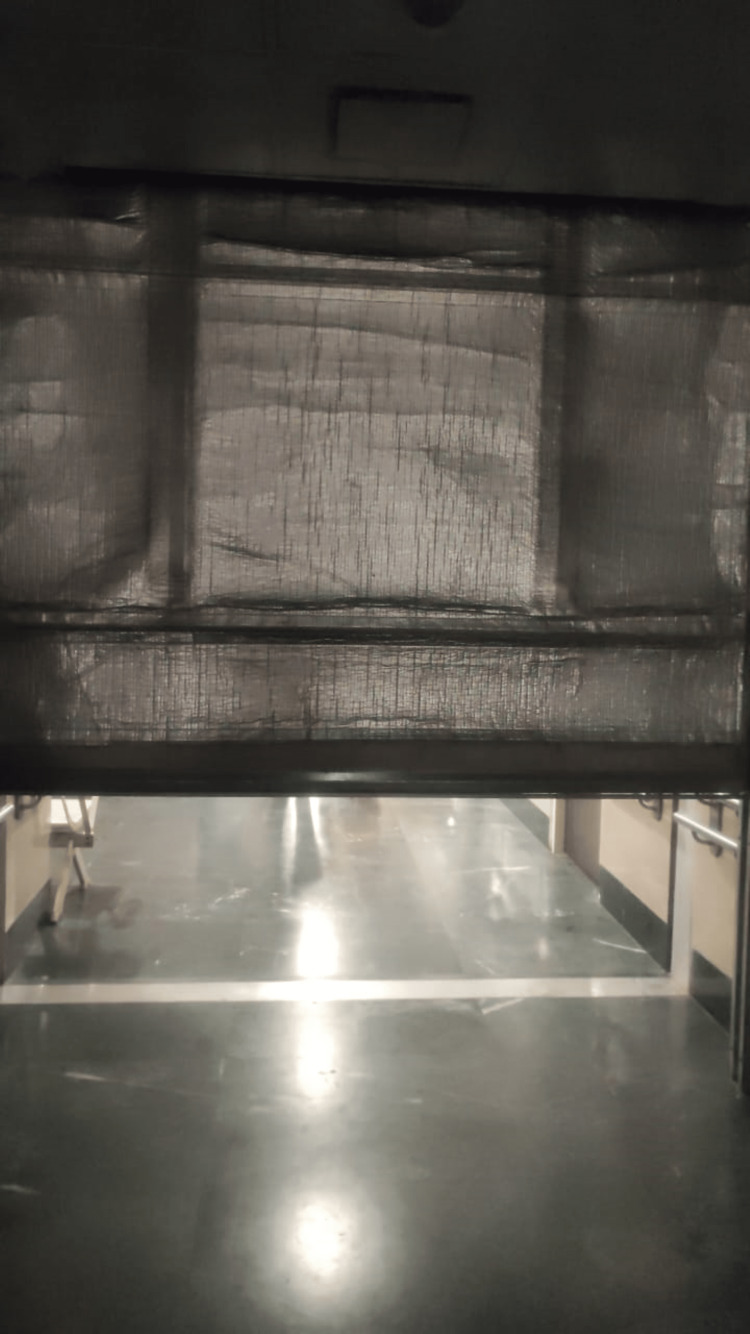
Fire curtain. A fire curtain helps in containing the fire in one compartment.

Additionally, smoke and fire curtains should be used, typically at the top of an elevator or stairway opening. These curtains can be manually or automatically positioned to restrict the smoke and fire at the source and stop it from spreading to other areas of the structure. In buildings with atriums or open lobbies that continue upward to multiple storeys, a horizontal smoke screen may aid in stopping smoke from moving. These smoke curtains also give fire extinguishing tools some extra time, reducing harm from increased smoke. We are using fire curtains in our hospital. These fire curtains are made of fire-resistant material, i.e., aluminum fiberglass fire-rated fabric (Figure 3).

Smoke dilution occurs when air is used to diffuse any combustion gases or smoke that may enter a non-fire environment while the air from that space is exhausted. Smoke dilution is also known as smoke extraction, purging, removal, or exhaust. This technique can be utilized by using leakage channels from an adjacent space to maintain appropriate gas and particle concentrations in a room susceptible to smoke penetration. Dilution can also help the fire department eliminate smoke after a fire has been put out. Doors may occasionally open, allowing smoke to enter spaces that were meant to be sealed off. By providing outside air to dilute the smoke, smoke penetrating spaces far from the fire can be expelled.

In pressurization, mechanical ventilation systems (fans) are used to create pressure differences across barriers with relatively high resistance to airflow. These pressure differences are used to manage the passage of smoke between compartments (i.e., small gaps). When space is pressurized, high-velocity airflows are created in construction cracks and small gaps surrounding closed doors, preventing smoke from backflowing through these holes. The pressurization method is frequently used in stairwells, elevator shaft pressurization, and zoned smoke control.

The airflow smoke control often refers to airflow through rather large openings. Airflow has frequently been used to control smoke from fires in subway, train, and highway tunnels. Smoke flow management requires high air flow rates.

Buoyancy is the term used to describe the venting of hot (buoyant) combustion gases through passive and fan-powered vents that are commonly found in the ceiling of big, open areas like atriums and covered malls.

To develop a good project, the engineer and the fire officers' team must work closely together. To effectively control smoke in the event of a fire, natural ventilation must also be considered in the smoke-control design. To create a functional, code-compliant, and economically viable design, designing a smoke-control project involves substantial experience and knowledge of numerous approaches and system combinations [8].

## Conclusions

The goal of smoke control is straightforward, i.e., to prevent smoke from spreading by various methods and control its direction by generating pressure differentials. The smoke is more dangerous as it causes asphyxial injury. To prevent these incidents, training programs and mock drills will give stakeholders the needed knowledge, skills, and practice they need to safeguard patients, employees, and the facility in the event of any such exigency.
